# Leiomyoma in a Renal Allograft

**DOI:** 10.1155/2016/8394942

**Published:** 2016-04-18

**Authors:** Yan Jun Li, Amila Rohan Siriwardana, James Lawrence Penn Symons, Gordon Francis O'Neill, Min Ru Qiu, Timothy John Furlong

**Affiliations:** ^1^Department of Renal Medicine, St. Vincent's Hospital, Victoria Street, Darlinghurst, NSW 2010, Australia; ^2^Department of Urology, St. Vincent's Hospital, Victoria Street, Darlinghurst, NSW 2010, Australia; ^3^Faculty of Medicine, University of New South Wales, Sydney, NSW 2052, Australia; ^4^Department of Anatomical Pathology, SydPath, St. Vincent's Hospital, Victoria Street, Darlinghurst, NSW 2010, Australia

## Abstract

Leiomyomas are smooth muscle tumours that are rarely found in the kidney. There is one report of a leiomyoma in a kidney transplant in a paediatric recipient. Here, we report an adult renal transplant recipient who developed an Epstein-Barr virus-positive leiomyoma in his allograft 15 years after transplantation. The patient was converted to everolimus for posttransplant immunosuppression management and there was no sign of progression over a year.

## 1. Introduction

Leiomyomas are benign neoplasms of smooth muscle origin that are commonly found in the uterus and rarely in the kidney. Immunosuppression is associated with the development of many smooth muscle tumours (SMT), most notably in the paediatric HIV population where SMT are the second most common neoplasm. Infection with Epstein-Barr virus (EBV) has also been associated with the development of SMT after transplantation. The rarity of this tumour type in the adult population, together with its varying organ localisation and clinical behaviour, presents a challenge for both diagnosis and management. Here, we report a patient who was diagnosed with an EBV-positive leiomyoma in his renal allograft 15 years after transplantation.

## 2. Case Description

A 54-year-old male with end-stage renal failure secondary to IgA nephropathy received a deceased donor renal transplant in 1999. He had received peritoneal dialysis for six years prior to transplantation. The posttransplant course was uneventful and graft function was excellent (serum creatinine concentration 90–100 *μ*mol/L). Maintenance immunosuppressive therapy was cyclosporine, prednisone, and azathioprine. Fifteen years after transplantation, the patient presented to the outpatient clinic for routine follow-up complaining of discomfort over his allograft. The serum creatinine concentration had increased to 120 *μ*mol/L. Urinalysis was normal.

**Figure 1:**
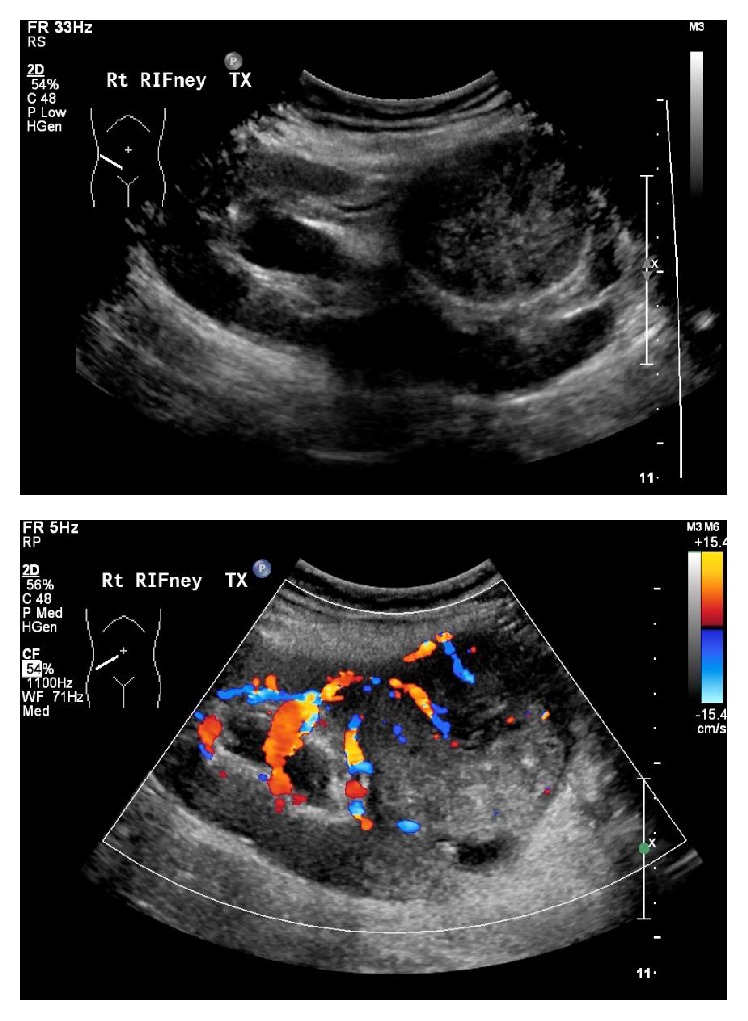
Ultrasound imaging showing a solid mass arising from the midpole of the renal allograft with internal vascularity.

**Figure 2:**
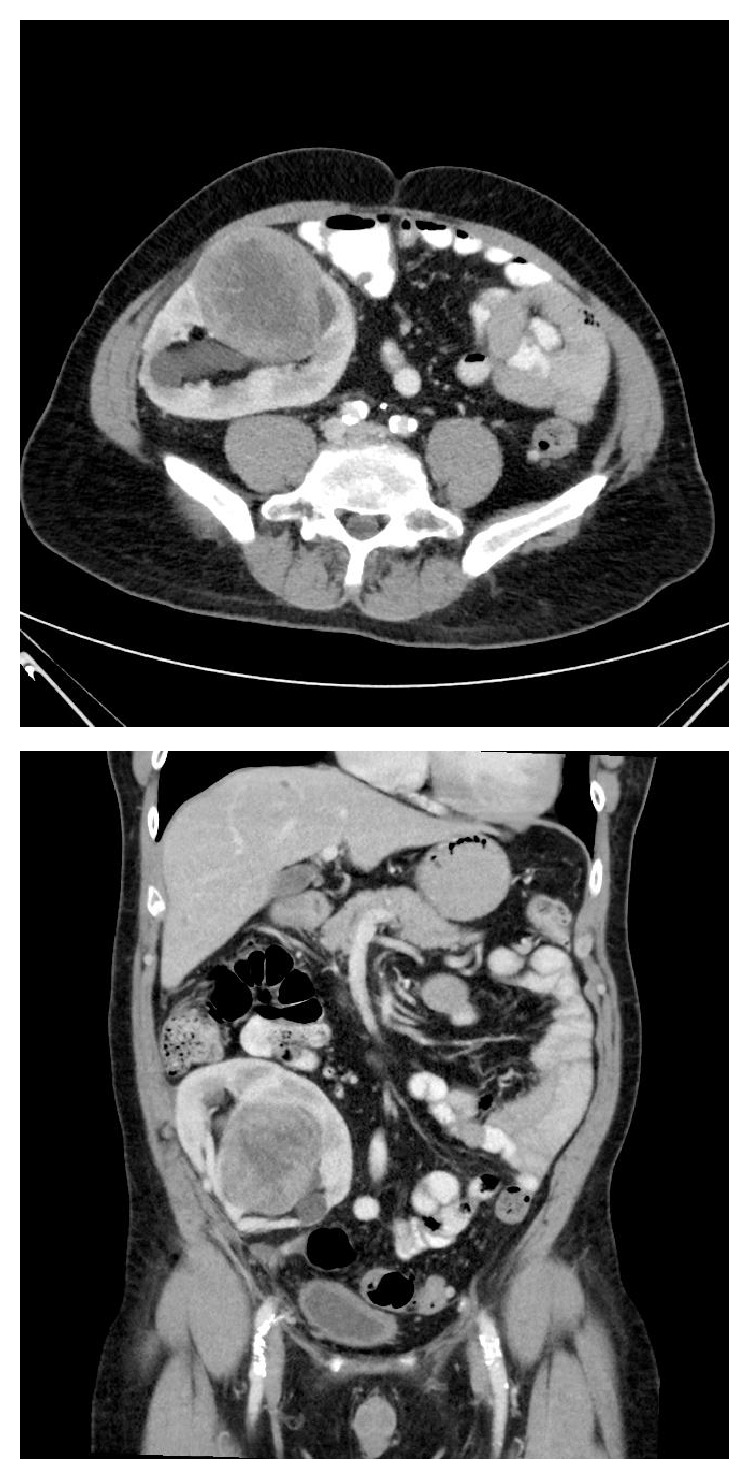
Axial and coronal contrast-enhanced computed tomographic images showing a heterogeneous, well circumscribed, soft tissue mass arising from the midpole of the renal allograft showing minor vascularity and less enhancement compared to surrounding parenchyma.

**Figure 3:**
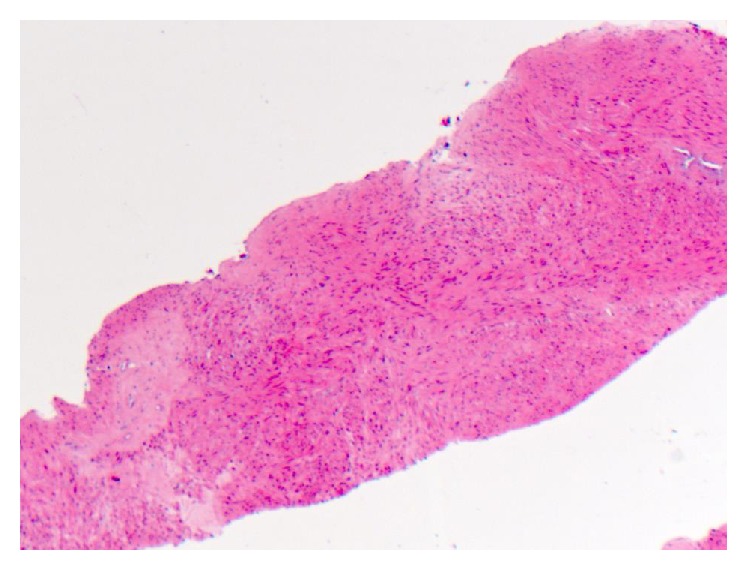
Haematoxylin and eosin staining showing spindle cell neoplasm composed of bundles of bland-looking spindle cells with eosinophilic cytoplasm (×40 magnification).

**Figure 4:**
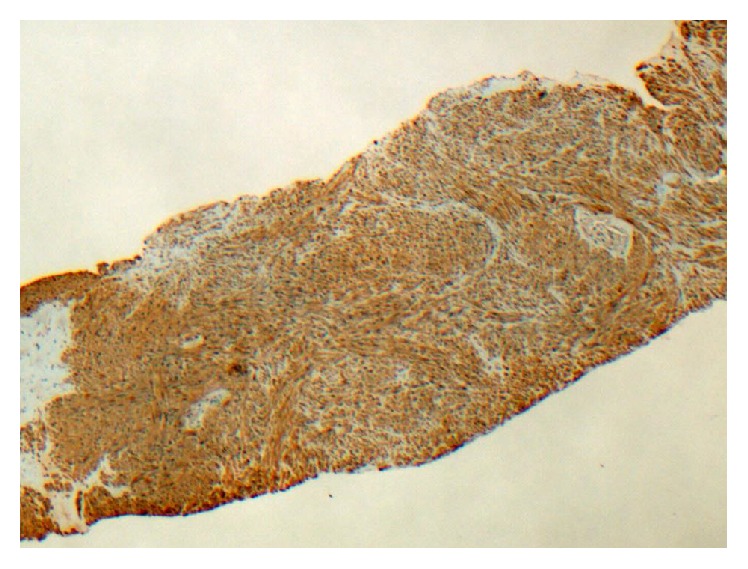
Immunohistochemical study: smooth muscle actin, positive (×40 magnification).

**Figure 5:**
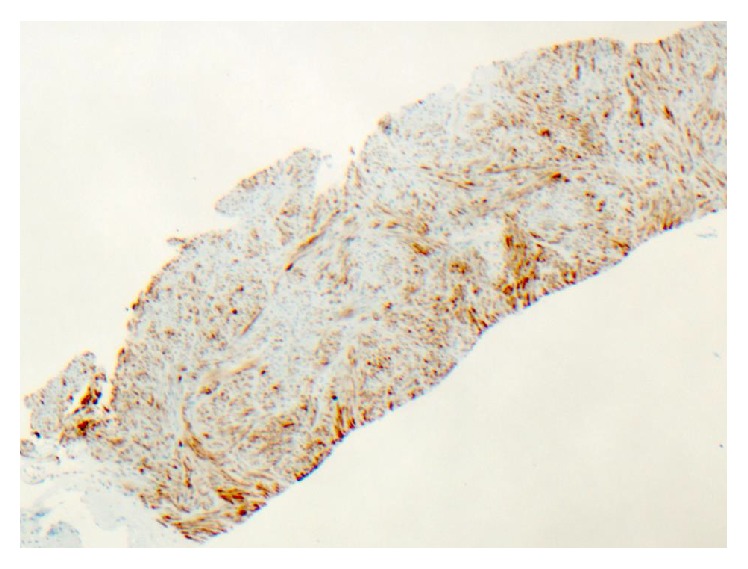
Immunohistochemical study: desmin, focally positive (×40 magnification).

**Figure 6:**
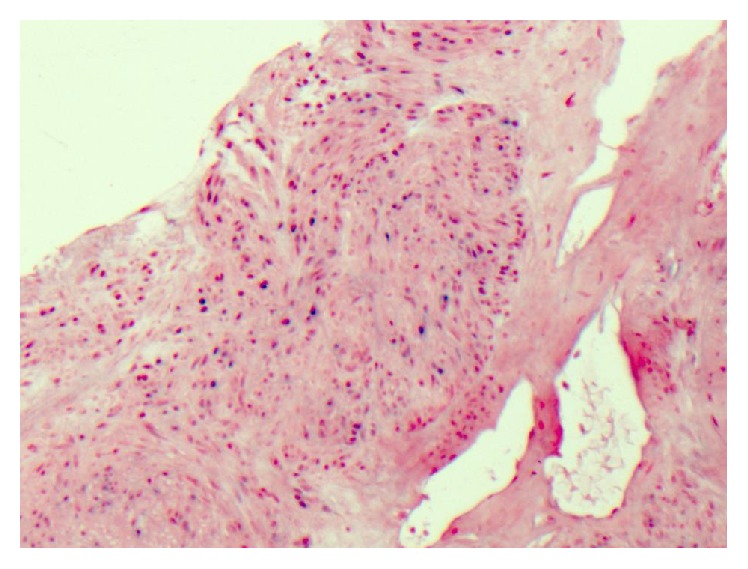
In situ hybridization for EBV early RNA: “blue dots” are positive nuclear staining (×100 magnification).

Transabdominal ultrasonography revealed an 8 cm solid vascular mass arising from the midpole of the transplanted kidney. It was partially obstructing the collecting system (Figure 1). Further characterisation with CT did not reveal local or distant disease (Figure 2). Sections from a core biopsy showed a spindle cell neoplasm composed of bundles of bland-looking spindle cells with eosinophilic cytoplasm without mitoses, necrosis, or nuclear pleomorphism (Figure 3). Immunohistochemical studies of the neoplastic cells were positive for SMA and desmin but negative for CD34, CD117, S100, and HMB45 (Figures 4 and 5). They were also positive for EBV RNA (Figure 6). The overall features were those of EBV-positive leiomyoma, a benign smooth muscle tumour.

Given the benign nature of the tumour, the relatively stable renal function, and the difficulty of total excision due to size and location, surveillance was deemed to be the most appropriate management. Treatment with cyclosporine and azathioprine was ceased and immunosuppression therapy was changed to everolimus. There was ongoing monitoring of renal function and graft morphology at three-monthly intervals. At the time of writing, 12 months following diagnosis, the patient's kidney transplant function was stable (serum creatinine concentration 108 *μ*mol/L). The tumour size was unchanged.

## 3. Discussion

Leiomyomas are benign neoplasms of smooth muscle origin, first described by Virchow in 1854 [1]. They are most commonly found in the uterus with a lifetime prevalence approaching 70–80% in women [2]. Renal leiomyomas are uncommon, with a 1971 autopsy series reporting an overall incidence of 5.2% [3]. Amongst tumours large enough to warrant surgical removal, their incidence is rarer still, with a recent review of over 1,000 nephrectomies noting an incidence of 0.3% of all* treated* tumours [4]. This would suggest that most of them are relatively indolent and do not result in nephrectomy.

In the kidney, leiomyomas may arise from the renal capsule, pelvis, or vascular smooth muscle. They are usually detected as an incidental renal mass on imaging. Less commonly, they are associated with haematuria and abdominal pain. Renal leiomyomas are benign with no reports of malignant transformation or metastasis. Unfortunately, however, they cannot be reliably distinguished from renal cell carcinomas or other malignant neoplasms based upon imaging features alone [5]. Given the well-known increased risk of malignancy following solid organ transplant (as a consequence of chronic immunosuppression), the existence of a solid renal lesion is always a cause of concern [6].

There is an association between immunosuppression and occurrence of EBV-positive SMT in both the paediatric AIDS population and adults after transplantation [7]. It is thought that EBV enters the smooth muscle cells through a CD21-positive receptor [8]. The argument for a causative association between EBV and SMT is based upon the observation that most SMT occurring in immune-compromised patients are EBV-positive whereas those in immune-competent patients are consistently EBV-negative [9, 10]. However, despite the high population seroprevalence of EBV, SMT are rare with only 18 case reports of leiomyomas following renal transplantation, one of which was located in the renal allograft [11, 12]. In this latter case, nephrectomy was performed without biopsy, with histopathology revealing an EBV-positive renal leiomyoma.

The diagnosis of renal leiomyomas can be challenging. In a recent review of 24 cases of renal leiomyoma, 15 were reclassified as angiomyolipoma, myolipoma, or medullary fibroma [13]. Histological examination of a renal leiomyoma reveals fusiform to spindle shaped cells with positive immunohistochemical staining for smooth muscle markers (desmin, caldesmon, and SMA) and negative staining for HMB-45 and cathepsin K. It should* not *reveal atypical nuclei, mitotic figures, or microscopic necrosis [13]. The differential diagnosis includes renal cell carcinoma, angiomyolipoma, leiomyosarcoma, and oncocytoma. Renal cell carcinomas are usually distinguished by the presence of perilesion invasion and positive staining for cytokeratin. Angiomyolipomas are fat-containing lesions with positive immunohistochemical staining for HMB-45. Leiomyosarcomas contain frequent mitotic figures, nuclear pleomorphism, and nuclear hyperchromatism. Because of the difference in outcome and management of these various lesions, a definitive histopathologic diagnosis is important.

The benign nature of renal leiomyomas means that surgical resection is curative. They are usually slow growing, locally invasive tumours without metastatic potential. Like renal oncocytomas, they are not innocuous, however, as they can continue to grow and disrupt the surrounding kidney tissue. Partial nephrectomy can be utilised for smaller peripheral masses of capsular or subcapsular origin. However, total nephrectomy remains the recommended treatment for larger central leiomyomas involving the collecting system. Nephrectomy in a renal transplant recipient would entail recommencement of dialysis. In this situation, conservative management with close surveillance should be considered.

There is an increased burden of cancer in renal transplant recipients. Management of malignancies in renal transplant recipients with mammalian target of rapamycin (mTOR) signalling pathway inhibitors, such as everolimus, has been recommended because of the antioncogenic effects [14]. mTOR inhibitors have been shown to reduce the incidence of malignancies and possibly cause regression of renal cell carcinoma, skin cancers, and Kaposi's sarcoma in transplant patients [15].

## 4. Conclusion

Renal leiomyomas are rare, with only 1 report of a leiomyoma within a renal allograft. Here, we describe a case of an adult patient who presented 15 years after transplantation with abdominal pain and was found to have a mass in his renal allograft. Given the long history of immunosuppression, malignancy was feared. Tissue for diagnosis was obtained with core biopsy. Histologic features were consistent with leiomyoma with the presence of smooth muscle spindle cells without necrosis, atypical nuclei, or mitotic figures. The leiomyoma was treated conservatively, with the patient switched from cyclosporine and azathioprine to the mTOR inhibitor everolimus. The tumour did not increase in size over the subsequent year and renal transplant function was stable.
